# Role of Acid‐Sensing Ion Channels 1a in the Regulation of Obesity and the Gut Microbiota

**DOI:** 10.1002/oby.70059

**Published:** 2025-10-26

**Authors:** Jane Shearer, Morris H. Scantlebury, Oghenefejiro Erome‐Utunedi, Anamika Choudhary, Jennifer A. Thompson, Christina Ohland, Kathy D. McCoy, Chunlong Mu

**Affiliations:** ^1^ Department of Biochemistry and Molecular Biology, Cumming School of Medicine University of Calgary Calgary Alberta Canada; ^2^ Alberta Children's Hospital Research Institute University of Calgary Calgary Alberta Canada; ^3^ Faculty of Kinesiology University of Calgary Calgary Alberta Canada; ^4^ Department of Pediatrics University of Calgary Calgary Alberta Canada; ^5^ Hotchkiss Brain Institute, Cumming School of Medicine University of Calgary Calgary Alberta Canada; ^6^ Department of Clinical Neuroscience, Cumming School of Medicine University of Calgary Calgary Alberta Canada; ^7^ Department of Physiology and Pharmacology, Snyder Institute Cumming School of Medicine Calgary Alberta Canada; ^8^ Department of Microbiology, Immunology, and Infectious Diseases, Cumming School of Medicine University of Calgary Calgary Alberta Canada

**Keywords:** acid‐sensing ion channel, gut microbiota, insulin resistance, obesity

## Abstract

**Objective:**

Acid‐sensing ion channels are proton‐activated ion channels predominantly found in the nervous system. They are well known to affect metabolic and neurological health, yet their role in obesity and gut physiology remains unclear. This study investigates how systemic deletion of *Asic1a* influences obesity, metabolic, and gut‐based outcomes.

**Methods:**

Employing male and female rats with systemic *Asic1a* deletion (*Asic1a* ^−/−^), metabolic, gut, and fecal microbiota (16S rRNA sequencing) measures were assessed following chow diet or high‐fat diet administration for 8 weeks. Fecal microbiota transplantation into germ‐free mice was carried out as a proof‐of‐concept approach to assess the gut microbiota's direct impact.

**Results:**

On a chow diet, *Asic1a* deletion resulted in significant gains in body weight, fat mass, glucose intolerance, and insulin resistance in both male and female rats compared to wild‐type controls. These effects were exacerbated with high‐fat diet administration. *Asic1a* ^−/−^ reshaped the gut microbiota, characterized by the enrichment of *Bacteroides* and *Akkermansia*. Microbiota transplantation from *Asic1a* ^−/−^ rats to recipient germ‐free mice increased body weight gain relative to those from wild‐type rats, implicating the potential role of gut microbiota.

**Conclusions:**

Results provide evidence that ASIC1a plays a role in regulating metabolic homeostasis and the gut microbiota impacting body composition.

**Study Importance**

- What is already known?
  ○ Acid‐sensing ion channels are proton‐gated cation channels that detect extracellular pH changes and visceral mechano‐sensation in the gastrointestinal tract.
  ○ Systemic knockout of *Asic1a* affects intestinal mechanical sensitivity leading to delays in gastric emptying.
- What does this study add?
  ○ Systemic knockout of *Asic1a* causes obesity and insulin resistance, impacts that are exacerbated by high‐fat diet administration.
  ○ *Asic1a* knockout alters gut physiology and fecal microbiota structure that contribute to obesity progression.
- How might these results change the direction of research?
  ○ Findings highlight the underrepresented role of the acid‐sensing ion channel in regulating metabolic homeostasis and gut microbiota composition.
  ○ Deciphering ASIC1a regulation could uncover new targets contributing to obesity pathophysiology.

## 1 Introduction

Obesity has emerged as a global health challenge, significantly elevating the risk of noncommunicable diseases such as type 2 diabetes and cardiovascular disease. Obesity is now understood to be more complex than an imbalance between energy intake/expenditure and results from a complex interplay between genetic predispositions and environmental influences. Both monogenic and polygenic forms of obesity have been identified, with key genes implicated in energy homeostasis and appetite regulation, including *LEP* (leptin), *MC4R* (melanocortin 4 receptor), *POMC* (pro‐opiomelanocortin), *LEPR* (leptin receptor), and *BDNF* (brain‐derived neurotrophic factor) [1]. However, genetics alone cannot account for the rapid rise in global obesity rates. Emerging evidence highlights the gut microbiota as a critical environmental factor that modulates host metabolism, insulin sensitivity, immune function, and intestinal barrier integrity [2, 3]. Therefore, deciphering how genetic factors interact with environmental cues such as diet, microbiota, and lifestyle is essential for understanding the pathogenesis of metabolic disorders and developing precision‐based therapies.

Proton‐gated acid‐sensing ion channels (ASICs) are members of the epithelial Na^+^ channel superfamily and are activated in response to extracellular acidosis in the central and peripheral nervous system [4]. ASICs include at least six isoforms: ASIC1a, ASIC1b, ASIC2a, ASIC2b, ASIC3, and ASIC4. ASICs function by assembling into homo‐ or hetero‐multimeric channels, and most (excluding ASIC1b) have been studied in the central nervous system [5]. Among them, the homomeric ASIC1a is particularly sensitive to pH changes, with acidification triggering neuronal firing [6]. Targeted knockdown of ASIC1a in the paraventricular nucleus of the hypothalamus of mice results in increased body weight gain and insulin resistance, highlighting the role of ASIC1a in metabolic dysregulation [7]. However, the role of ASICs in gut and metabolic disturbances such as obesity and insulin resistance remains poorly understood.

Beyond the central nervous system, ASICs also regulate peripheral physiological activities, including those in the gastrointestinal tract. In the gut, ASIC1a is expressed in the peripheral sensory neurons of extrinsic primary afferent fibers originating from the dorsal root and nodose ganglia [8]. Systemic loss of ASIC1a increases intestinal mechanical sensitivity and doubles the gastric emptying time in mice [9, 10]. Given the tight interaction between gut microbiota and gastrointestinal motility [11], it is of interest to investigate how *Asic1a* deletion affects the gut microbiota.

We hypothesized that ASIC1a affects obesity progression and the development of insulin resistance. Here, we use whole body *Asic1a* knockout rats to uncover the role of ASIC1a in adiposity, insulin sensitivity, and gut physiology, including the gut microbiota. To determine whether the altered gut microbiota contributes to obesity, a pilot fecal microbiota transplantation trial was conducted in germ‐free mice. These findings shed light on the underappreciated role of proton‐gated acid‐sensing ion channels in modulating metabolic homeostasis and shaping gut microbial composition.

## 2 Methods

### 2.1 Animals

All experimental procedures were approved by the Health Sciences Animal Care Committee at the University of Calgary in accordance with the guidelines set forth by the Canadian Council on Animal Care (protocol #AC21‐0014). Sprague–Dawley females were bred in‐house, in full compliance with the ethical protocol. Animals were housed at a constant temperature (20°C–22°C) on a 12‐h light/dark cycle at the specific pathogen‐free facility of the Health Sciences Animal Resource Centre, University of Calgary, Ontario, Canada.

### 2.2 Generation of *Asic1a* ^−/−^ Animals

*Asic1a* knockout rats were generated through a CRISPR/Cas9 protocol [12]. Briefly, recipient females (Sprague‐Dawley rat, 175–200 g body weight) were administered luteinizing releasing hormone 3 days prior to mating to trigger ovulation. Donor females (Sprague‐Dawley rat, 3–4 weeks of age) were superovulated 3 days prior to breeding with stud males (Sprague‐Dawley rat, 226–250 g body weight). Recipient females were simultaneously mated with vasectomized males (Sprague‐Dawley rat, 12 weeks of age) to produce pseudo‐pregnant females. At 0.5 days post coitum, superovulated pregnant female rats were euthanized to harvest fertilized eggs. The CRISPR/Cas9 gene was delivered to the embryos by pronuclear injection. Around 18–25 embryos were subsequently transferred bilaterally into the oviduct of each pseudo‐pregnant female. At weaning, pups were ear‐notched and the tissue samples collected for genotyping to identify CRISPR knockout offspring. Validation of *Asic1a* knockout was confirmed by Western blot (Figure S1). The breeding protocol generated a colony of rats with a 20 bp deletion, yielding the ASIC1a protein inactive.

### 2.3 Diets

Starting from weaning (postnatal day 21), both female and male rats were provided either a standard chow diet (CD; 13% of kilocalories from fat, 62% of kilocalories from carbohydrate, and 24% of kilocalories from protein, Cat# PicoLab Rodent Diet 5053, LabDiet) or a high‐fat diet (HFD; 45% of kilocalories from fat, 35% of kilocalories from carbohydrate, and 20% of kilocalories from protein, Cat# D12451, Research Diets). The primary source of fat in the HFD was lard. Animals have access to water and feed ad libitum. Body weight was monitored weekly. Food intake was recorded every 3 days.

### 2.4 Body Composition

Body mass and composition (fat, lean, and fluid mass) were measured using a Minispec LF90II spectrometer (Bruker) with a rat probe. All measures were normalized to body weight.

### 2.5 Glucose and Insulin Tolerance Tests

Glucose (OGTT) and insulin tolerance (ITT) tests were performed following the recommended guidelines [13] and as previously described [14]. The OGTT was performed at postnatal day 56. Following a 6‐h fast (7 a.m.–1 p.m.), blood glucose was measured via tail nick sample using a portable touch glucose meter (One Touch Verio Pro+ glucometer, LifeScan). Glucose was administered via oral gavage at a 2.0 g/kg body weight load followed by blood glucose measurements at 0, 15, 30, 60, 120, and 180 min. An ITT was subsequently performed on postnatal day 63 following a 6‐h fast (7 a.m.–1 p.m.). Briefly, 1.0 IU/kg body weight of insulin (Humulin, Eli Lilly) was administered subcutaneously and glucose concentration was measured from the tail vein at 0, 15, 30, 60, 120, and 180 min following injection. Total area under the curve (AUC) and incremental AUC (iAUC) above baseline were calculated. The glucose fall rate with insulin was calculated and reported for the first 30 min.

### 2.6 Blood Measurement

At day 73, blood was collected from the femoral vein and used to measure lactate using a Stat Profile pHOx Ultra analyzer (Nova Biomedical) following the manufacturer's instructions. On the day of sacrifice, blood glucose and ketones were measured using a glucometer and ketone meter (FreeStyle Precision Neo), respectively.

### 2.7 Fecal Pellet Output

Fecal pellet output was monitored at day 37 as a proxy of intestinal transit rate. Briefly, animals were transferred into a new cage with new bedding materials. Fecal pellet number in 24 h was counted per cage and then divided by the number of animals to calculate the fecal output per rat. Total pellet weight was also measured.

### 2.8 Fecal Genomic DNA Extraction and 16S rRNA High‐Throughput Sequencing

Bacterial genomic DNA was extracted and sequenced as described previously [15]. A total of 100 mg of fecal sample was extracted with a DNeasy PowerSoil Pro Kit (QIAGEN). The quantity and quality of DNA were quantified using the high‐sensitivity dsDNA Qubit Kit (Invitrogen) and NanoDrop Spectrophotometer, respectively. The V3‐V4 region of the 16S gene was paired‐end sequenced using an Illumina MiSeq platform with the MiSeq V3 600 cycle sequencing kit. The sequencing was performed in the Centre for Health Genomics and Informatics at the University of Calgary. Data of amplicon sequence variant (ASV) were produced using DADA2 version 1.8 [16]. ASV reads were normalized with centered log‐ratio methods [17]. The R package *vegan* was used to analyze microbial alpha (Shannon index) and beta diversity (permutational multivariate analysis of variance, PERMANOVA). LEfSe was used for identifying discriminant features between groups [18]. Linear discriminant analysis (LDA) effect size > 2 indicates significant features.

### 2.9 Fecal Microbiota Transplantation

A pilot, proof‐of‐concept fecal microbiota transplantation trial was performed following standard protocols by the International Microbiome Centre at the University of Calgary [19]. Germ‐free male mice were utilized for this study. Briefly, 4‐week‐old male C57BL/6 germ‐free mice were used for transplantation. Stool collected from male wild‐type or *Asic1a* ^−/−^ rats fed CD at postnatal day 70 was pooled and homogenized in 20% glycerol with l‐cysteine hydrochloride based on stool weight (0.2 g stool with 2 mL glycerol). Prior to gavage, the supernatant was mixed with prereduced phosphate‐buffered saline (PBS) in an anaerobic chamber to create a homogeneous solution. Then 200 μL of the PBS‐stool solution was orally gavaged into male germ‐free mice twice on days 1 and 3 of the experiment. Body weight was monitored at baseline and 7, 14, and 21 days after gavage. At 21 days post gavage, after 6 h of fasting, an OGTT was performed at 0, 15, 30, 60, and 90 min following oral glucose administration.

### 2.10 Statistics

Normality was assessed with a Kolmogorov–Smirnov test. For the time‐series data (body weight gain, glucose, and insulin tolerance), a two‐way ANOVA with repeated‐measure analysis was applied to compare the effects of age/time and treatment (diet or genotype). Males and females were analyzed separately where there was a main effect of sex or interaction between sex and treatment. Data on blood biochemistry and fecal output were analyzed using two‐way ANOVA (sex × genotype). For the ITT, differences in glucose fall rate at the first 30 min between wild type and knockout were compared using Student's *t* test. Data on microbial diversity at a single time point were analyzed using a two‐tailed Student's *t* test. Significant differences are indicated by *p < 0.05, **p < 0.01, ***p < 0.001, ****p < 0.0001. Values of *p* < 0.05 were considered significant, while *p* < 0.1 indicated a trend toward a significant effect.

## 3 Results

### 3.1 *Asic1a* Deletion Leads to Obesity and Insulin Resistance

**FIGURE 1:**
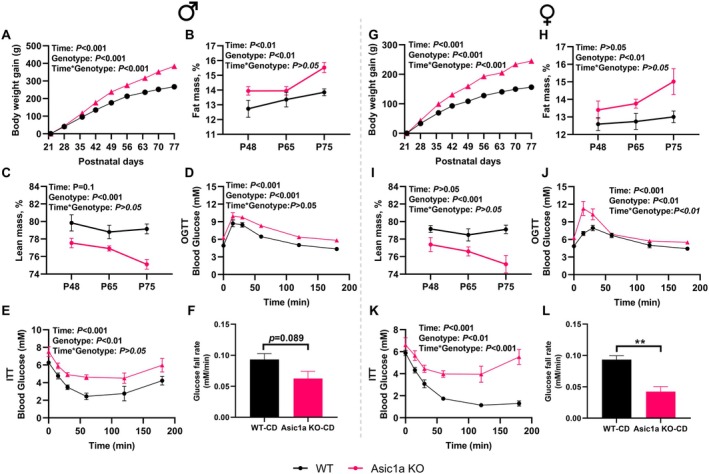
Effects of *Asic1a* deletion on animal growth and metabolic outcomes in male and female rats. (A) Body weight gain, (B) fat mass, (C) lean mass, (D) oral glucose tolerance test (OGTT), (E) insulin tolerance test (ITT), and (F) glucose fall rate in the first 30 min of insulin tolerance test (*n* = 7 for wild type, *n* = 5 for knockout). (G–L) Same parameters in females (*n* = 5 for both wild type and knockout). Data are presented as mean ± SEM. In panels A–E and G–K, 2‐way ANOVA with repeated‐measure analysis was applied to compare the effects of time and genotype. In panels F and L, statistical difference was analyzed by 2‐tailed Student's *t* test. **p < 0.05. Asic1a KO, acid‐sensing ion channels 1a knockout; CD, chow diet; WT, wild type. [Color figure can be viewed at wileyonlinelibrary.com]

To investigate growth and the metabolic phenotype, wild‐type and *Asic1a* ^−/−^ rats were monitored until 11 weeks of age. Male *Asic1a* ^−/−^ rats fed CD gained more body weight (*p* < 0.001, Figure 1A), characterized by a higher fat mass (*p* < 0.01, Figure 1B) and lower lean mass (*p* < 0.001, Figure 1C) than wild‐type rats throughout the experimental period. *Asic1a* ^−/−^ rats exhibited a greater glucose response to the OGTT (*p* < 0.001, Figure 1D) with significantly higher total AUC (*p* < 0.001, Figure S2), while the iAUC was increased in knockout animals but not significantly (*p* > 0.05, Figure S2), indicating increased glucose responses were mainly driven by elevated fasting glucose with *Asic1a* deletion. *Asic1a* ^−/−^ rats exhibited greater insulin resistance (*p* < 0.01, Figure 1E). The glucose fall rate after insulin injection tended to be decreased in males (*p* = 0.089) by *Asic1a* deletion (Figure 1F). A similar phenotype was observed in *Asic1a* ^−/−^ female rats, as shown by increased body weight gain (*p* < 0.001, Figure 1G) and fat mass (*p* < 0.01, Figure 1H) and decreased lean mass (*p* < 0.001, Figure 1I). Glucose response in OGTT (*p* < 0.01, Figure 1J) was also greater with *Asic1a* deletion in females. The total AUC of glucose response was significantly higher in *Asic1a* ^−/−^ rats (*p* < 0.001), while the iAUC was increased but not significantly in males (*p* > 0.05, Figure S2). The insulin resistance (*p* < 0.01, Figure 1K) was also greater with *Asic1a* deletion. The glucose fall rate after insulin injection was significantly decreased in females with *Asic1a* deletion (*p* = 0.002, Figure 1L). Collectively, the data provide compelling evidence that loss of ASIC1a leads to obesity, accompanied by impaired insulin sensitivity. Monitoring of food intake suggests that *Asic1a* ^−/−^ tended to increase food intake in both CD and HFD conditions (Figure S3A,B).

Blood analysis showed that *Asic1a* ^−/−^ decreased blood ketone levels (*p*_*Genotype*_ < 0.001, Figure S4A) in both males and females and had no effects on lactate (*p*_*Genotype*_ = 0.211, Figure S4B). There were no sex differences in blood ketone and lactate (*p*_*Sex*_ > 0.05, Figure S4A,B).

Measurement of intestinal behavior showed no impact of sex or genotype on the number of fecal pellets excreted in 24 h (Figure S4C). However, *Asic1a* ^−/−^ increased the fecal output in both males and females (*p*_*Genotype*_ = 0.001, Figure S4D). Females secreted less fecal mass than males (*p*_*Sex*_ = 0.006, Figure S4D).

### 3.2 *Asic1a* Deletion Exacerbates HFD‐Induced Obesity and Insulin Resistance

**FIGURE 2:**
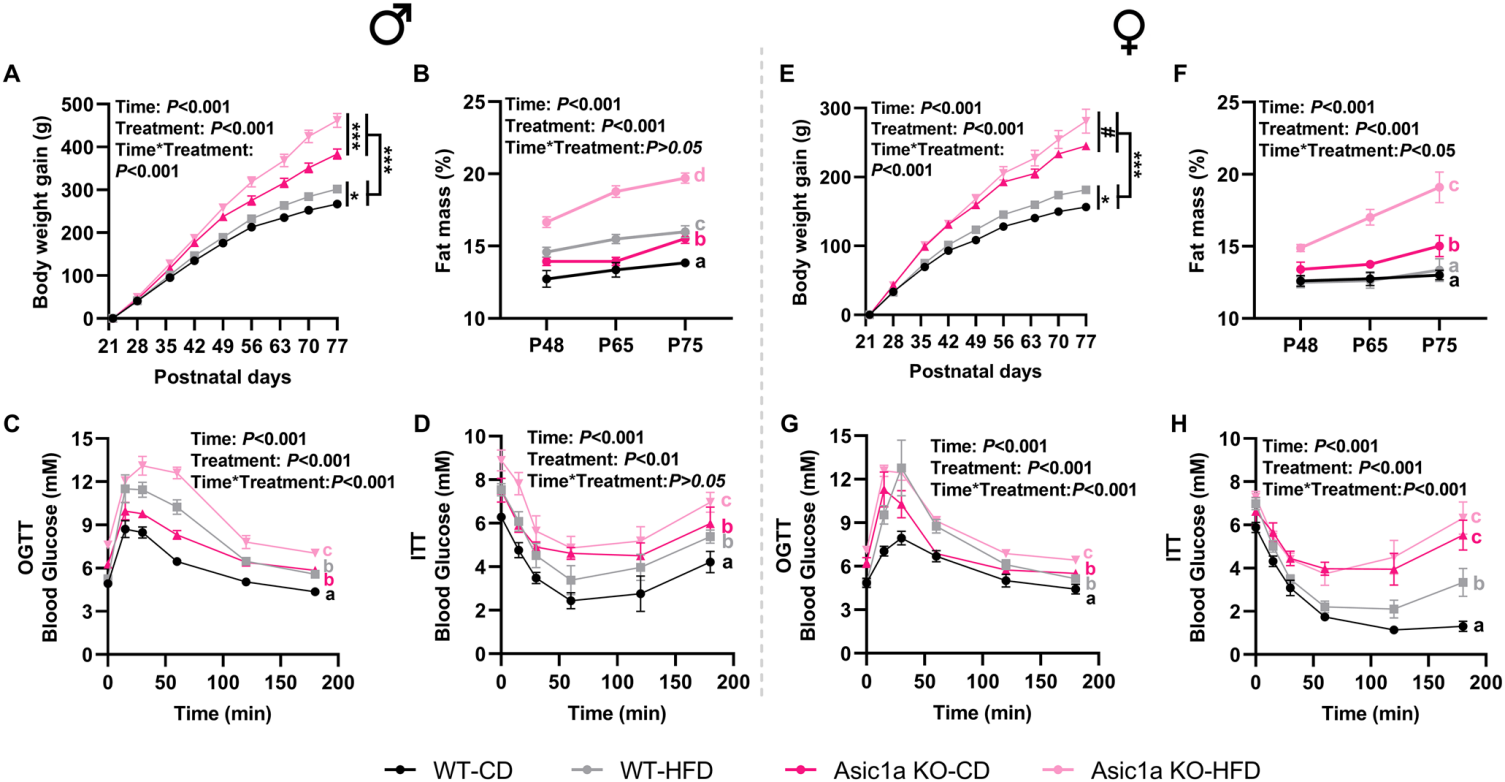
Effects of high‐fat diet on metabolic outcomes in male and female Asic1a‐null rats. (A) Body weight gain, (B) fat mass, (C) oral glucose tolerance test (OGTT), and (D) insulin tolerance test (ITT) in males (*n* = 5, 7, 5, 5 for chow diet‐fed wild type, high‐fat diet‐fed wild type, chow diet‐fed knockout, and high‐fat diet‐fed knockout, respectively). (E–H) Same parameters in females (*n* = 5 each for chow diet‐fed wild type, high‐fat diet‐fed wild type, chow diet‐fed knockout, and high‐fat diet‐fed knockout). Data are presented as mean ± SEM. Statistical differences were analyzed by 2‐way ANOVA with repeated‐measure analysis. In panels A and E, 0.05 < ^#^ *p* < 0.1, *p < 0.05, ***p < 0.001. In panels B–D and F–H, different letters indicate significant difference, *p* < 0.05. Asic1a KO, acid‐sensing ion channels 1a knockout; CD, chow diet; HFD, high‐fat diet; WT, wild type. [Color figure can be viewed at wileyonlinelibrary.com]

**FIGURE 3:**
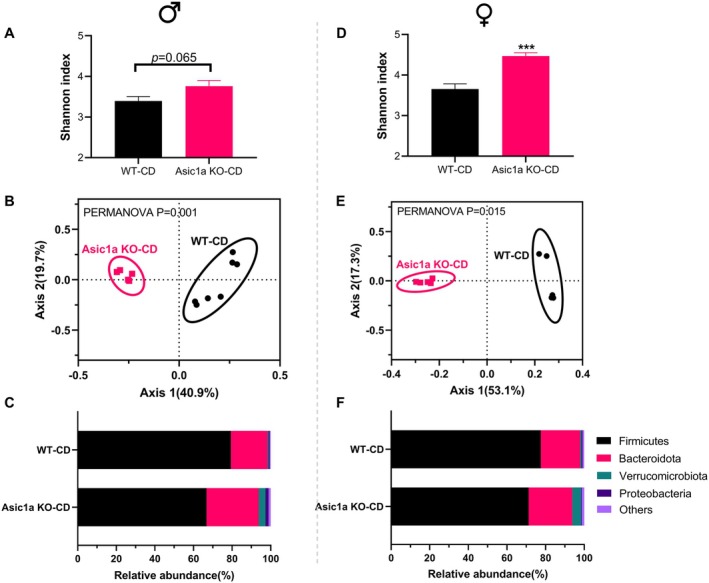
Effects of *Asic1a* deletion on fecal microbiota. (A) Shannon index, (B) Bray‐Curtis distance‐based principal coordinate analysis, and (C) phylum‐level composition in males (*n* = 7 for wild type, *n* = 5 for knockout). (D–F) Same readouts in females (*n* = 5 for both wild type and knockout). In panels A and D, data are presented as mean ± SEM. Statistical difference was analyzed by 2‐tailed Student's *t* test. ***p < 0.001. In panels B and E, between‐group difference was tested by permutational multivariate ANOVA. In panels C and F, data are presented as mean relative abundance. Asic1a KO, acid‐sensing ion channels 1a knockout; CD, chow diet; PERMANOVA, permutational multivariate ANOVA; WT, wild type. [Color figure can be viewed at wileyonlinelibrary.com]

To assess the impact of ASIC1a in response to overnutrition, the rats were provided with HFD with 45% of kilocalories from fat. In males fed HFD, *Asic1a* deletion resulted in the highest body weight gain and fat mass relative to wild type (Figure 2A,B). Interestingly, rats with *Asic1a* deletion with CD gained more weight than HFD‐fed wild‐type rats (*p* < 0.001, Figure 2A). HFD feeding further provoked glucose intolerance (Figure 2C) and insulin resistance (Figure 2D) in *Asic1a* ^−/−^ rats. CD‐fed *Asic1a* ^−/−^ rats and HFD‐fed wild‐type rats had a comparable level (*p* > 0.05) of glucose tolerance and insulin resistance, which were both greater than CD‐fed wild‐type rats (*p* < 0.05, Figure 3C,D). In females, HFD feeding in *Asic1a* ^−/−^ further increased body weight gain (Figure 2E), fat mass (Figure 2F), glucose intolerance (Figure 2G), and insulin resistance (Figure 2H) as observed in males. In terms of insulin resistance, CD‐fed *Asic1a* ^−/−^ rats showed a greater degree than HFD‐fed wild‐type rats (*p* < 0.05). Given this, it is clear that *Asic1a* deletion leads to pronounced metabolic disturbances manifesting as obesity, glucose intolerance, and insulin resistance that are further exacerbated by HFD.

### 3.3 Deletion of *Asic1a* Significantly Reshapes Gut Microbiota Composition

To investigate whether the phenotype induced by *Asic1a* deletion is associated with changes in gut microbiota, we analyzed microbial composition in CD‐fed rats. In males, *Asic1a* deletion tended to increase alpha diversity, as measured by the Shannon index (*p* = 0.066; Figure 3A). Principal coordinate analysis based on Bray‐Curtis distances revealed a marked shift in overall microbial structure in *Asic1a*‐deficient rats (PERMANOVA *p* = 0.001; Figure 3B).

Although no significant changes were observed at the phylum level, *Asic1a* ^−^/^−^ rats exhibited numerically higher abundances of Bacteroidota and Verrucomicrobiota compared to wild‐type controls (*p* > 0.05; Figure 3C). In females, *Asic1a* deletion led to a significant increase in microbial diversity (*p* < 0.001; Figure 3D) and a distinct alteration in microbial structure (PERMANOVA *p* = 0.015; Figure 3E). The phylum‐level composition was similar between males and females (Figure 3F). However, *Asic1a* ^−/−^ decreased the Firmicutes to Bacteroidota ratio in both males and females (*p*_*Genotype*_ = 0.025, *p*_*Sex*_ = 0.93, Figure S5).

### 3.4 *Asic1a* Deletion Had a Prominent Impact on Gut Microbiota Structure and Composition

**FIGURE 4:**
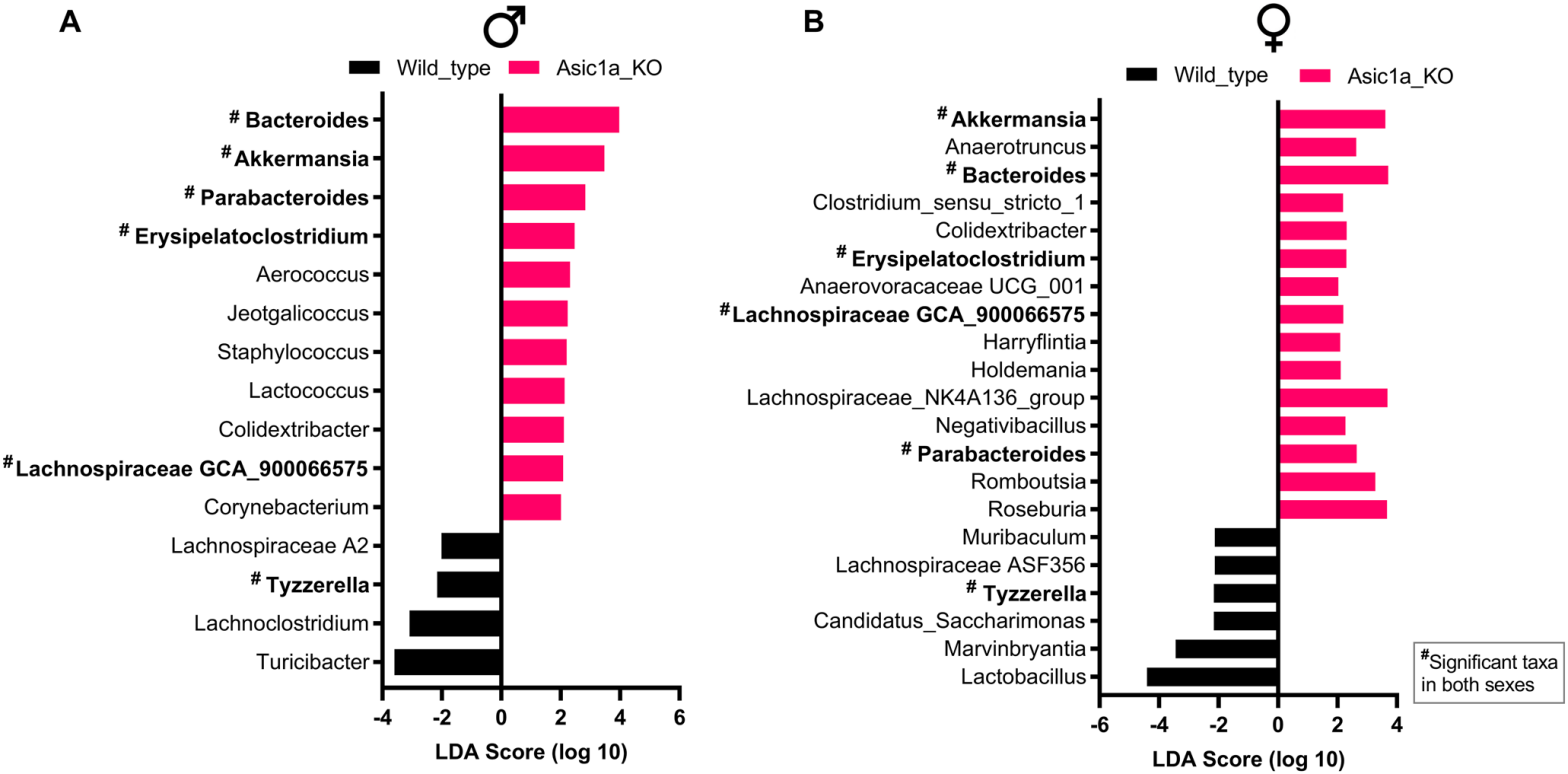
Discriminant microbial genera in (A) males and (B) females. Statistical difference was analyzed with the linear discriminant analysis (LDA) effect size. LDA > 2 indicates significant features. ^#^Significant taxa affected by *Asic1a* ^−/−^ in both sexes. Asic1a KO, acid‐sensing ion channels 1a knockout. [Color figure can be viewed at wileyonlinelibrary.com]

At the lower taxonomy level, several taxa were altered with *Asic1a* deletion. Based on the threshold of linear discriminant analysis score of 2, differential impacts were observed for males and females. In males, a total of 32 features were significantly affected (*p* < 0.05, Figure S6A). *Asic1a* deletion enriched Verrucomicrobiales, Staphylococcaceae, *Bacteroides*, *Parabacteroides*, *Lactococcus*, *Staphylococcus*, and *Akkermansia* and decreased *Turicibacter* and *Lachnoclostridium* (Figure 4A and Figure S6A).

In females, a total of 51 features were affected (*p* < 0.05, Figure 4B and Figure S6B). *Asic1a* deletion enriched Verrucomicrobiales, *Bacteroides*, *Parabacteroides*, *Roseburia*, *Romboutsia*, and *Akkermansia* and decreased Staphylococcaceae, *Muribaculum*, *Lactobacillus*, and *Marvinbryantia* (Figure 4B and Figure S6B). Overall, at the genus level, a total of six genera, including *Akkermansia, Bacteroides*, *Parabacteroides*, *Erysipelatoclostridium*, *Tyzzerella*, and Lachnospiraceae GCA_900066575, showed consistent directions of change in both males and females (Figure 4), while alterations of other taxa were sex‐specific with more impact observed in females (Figure 4). These observed shifts suggest that ASIC1a plays a previously underappreciated role in maintaining microbial homeostasis.

### 3.5 Fecal Microbiota Transplantation Partially Recapitulates the Metabolic Phenotype

**FIGURE 5:**
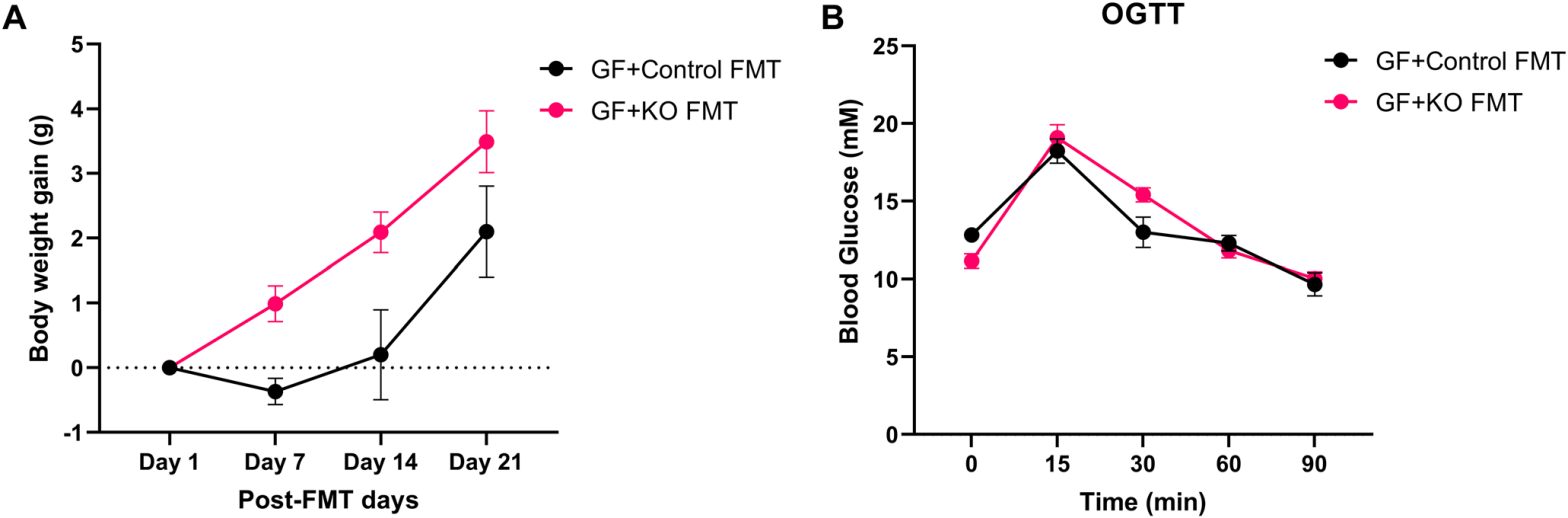
Effect of fecal microbiota transplantation on growth and metabolic outcome in germ‐free mice. (A) Body weight gain. (B) Oral glucose tolerance test. *n* = 3 and 9 for control and *Asic1a* knockout gavage groups, respectively. Data are presented as mean ± SEM. FMT, fecal microbiota transplant; GF, germ‐free; KO, knockout; OGTT, oral glucose tolerance test. [Color figure can be viewed at wileyonlinelibrary.com]

To further understand whether the altered microbiota is involved in the observed metabolic phenotype, a pilot trial using fecal microbiota transplantation was performed in a germ‐free facility. The transplantation of fecal suspension from *Asic1a* ^−/−^ rats significantly increased body weight gain in the 3‐week period compared to controls (*p* = 0.009, Figure 5A). Although a significant difference was not observed for glucose tolerance, the transplantation increased blood glucose concentration at 30 min post gavage (*p* < 0.05, Figure 5B). Therefore, fecal microbiota transplantation partially recapitulates the metabolic phenotype seen with *Asic1a* deletion.

## 4 Discussion

While ASICs are well characterized in the central nervous system, their role in obesity and metabolic syndrome remains largely unexplored. In this study, we present compelling evidence that systemic deletion of *Asic1a* predisposes rats to an obese phenotype characterized by increased fat mass and insulin resistance. Further, our findings suggest that part of this metabolic disruption is mediated through alterations in gut microbiota composition, highlighting a previously unrecognized interaction between ASIC1a, host metabolism, and microbial ecology.

The relevance of ASICs in metabolic regulation remains elusive. Supporting the observed metabolic alterations shown here, targeted knockdown of murine ASIC1a in the paraventricular nucleus of the hypothalamus increases body weight gain and food intake and provokes glucose intolerance and insulin resistance, with the effects being worse following HFD administration [7]. The elevated food intake observed following systemic *Asic1a* knockout in the present study, as well as in the hypothalamic‐specific knockout model [7], may partly explain the associated increase in body weight gain. In contrast, systemic knockout of *Asic3* leads to less body weight gain and improved insulin sensitivity in aging mice, an effect that is associated with upregulated expression of phosphorylated Akt in white adipose tissue [20]. Although the underlying mechanisms remain largely undefined, emerging evidence suggests that ASICs may exert distinct and context‐dependent effects within the peripheral nervous system. These differential actions are exemplified by their involvement in specific sensory pathways, such as the activation of gastroesophageal mucosal receptors [9], and the roles of specific ASIC isoforms in modulating key aspects of metabolism. Additionally, systemic deletion of *Asic1a* has been found to cause hypertension [21], reduce body temperature, and suppress daily rhythms of thyroid‐stimulating hormone‐releasing hormone mRNA in the hypothalamus that may contribute to altered metabolism [22]. ASIC1a is also a critical regulator of mitochondrial function as *Asic1a* knockout neurons show an impairment of mitochondrial permeability transition and increase oxidative stress [7], highlighting the importance of ASIC1a in metabolic health.

Deletion of *Asic1a* leads to alterations in fecal microbiota, including increased microbial α‐diversity and shifts in community structure, further suggesting a regulatory role for ASIC1a in shaping the gastrointestinal environment. As a proton‐gated channel in the gut, ASIC1a confers protective effects by transducing luminal acidification signals into bicarbonate secretion to maintain mucosal homeostasis [8]. Given that pH is a leading physiological factor affecting gut microbiota distribution [23], the inability to sense acid due to *Asic1a* deletion may impair bicarbonate production, thereby diminishing the gut's ability to counteract excessive acid production. Similarly, another study showed that loss of the acid‐sensing G protein‐coupled receptor 65 in intestinal epithelial cells led to gut microbiota alterations and a reduction in antimicrobial peptides that predisposed the mice to dextran sodium sulfate‐induced colitis [24]. Collectively, these findings highlight the underappreciated role of ion channels in the regulation of gut physiology.

The impact of *Asic1a* deletion exhibited sex‐dependent effects, with more pronounced alterations observed in females. Given that all animals were maintained on identical diets and exhibited similar levels of glucose intolerance, the observed sex‐specific differences in gut microbiota composition may reflect underlying differences in hormonal regulation and metabolic pathways, both of which are known to extensively interact with the gut microbiome [25, 26].

At the taxa level, *Asic1a* ^−/−^ decreased the Firmicutes to Bacteroidota ratio in both sexes. The ratio has long been debated as a potential biomarker of gut dysbiosis [27]. However, there are wide discrepancies in the direction of change across studies. For example, the Firmicutes to Bacteroidota ratio has been reported to increase in HFD‐induced obesity in both rats [28, 29] and mice [30, 31]. The decrease in this ratio observed alongside increased weight gain following *Asic1a* knockout suggests that ASIC1a may influence obesity through mechanisms distinct from those induced by HFD. Furthermore, factors such as experimental design, methodological differences, individual variability, and potential change in acidity due to ASIC type may complicate direct comparisons across studies. Results of the present study align with previous findings in individuals with overweight and obesity [32, 33], which demonstrate similar decreases in the Firmicutes to Bacteroidota ratio. This concordance supports the hypothesis that shifts in the relative abundance of these dominant bacterial phyla may be mechanistically linked to host metabolic dysregulation, potentially contributing to increased energy harvest, glucose intolerance, adiposity, and the pathophysiology of obesity.

We show *Asic1a* ^−/−^ enriched *Bacteroides* and Verrucomicrobiales and its predominant genus *Akkermansia* in both male and female rats. It is interesting to note that both *Bacteroides* and *Akkermansia* encompass species that are major mucin‐degrading bacteria [34, 35]. *Akkermansia* is well known to utilize mucin as its sole carbon and nitrogen source for survival [36, 37]. Verrucomicrobiales and *Akkermansia* species, such as *Akkermansia muciniphila* , have been reported to exert favorable impacts on obesity and type 2 diabetes [37]. However, excessive consumption of mucin by overcolonized *A. muciniphila* may also compromise the gut barrier, acting to promote disease in the context of colitis and colorectal cancer [38]. It should be noted that *A. muciniphila* affects the gut barrier in a strain‐specific manner, suggesting a complex underlying interaction [39]. Microbiota‐mucus interactions are fundamental to maintaining epithelial homeostasis. Disruption of this interaction can compromise the intestinal barrier and contribute to inflammatory bowel disease, metabolic syndrome, and even central nervous system disorders [40, 41, 42]. This highlights the need to investigate how ASIC1a regulates microbiota–mucus interactions and whether this influences metabolic outcomes, such as insulin resistance and obesity.

Our pilot trial involving fecal microbiota transplantation provides additional evidence for a causal role of ASIC1a‐induced alterations in the gut microbiota in the development of obesity. This aligns with previous findings showing that transplanting fecal microbiota from individuals with obesity into germ‐free mice can transfer obesity and its related traits including insulin resistance, thereby supporting a causal involvement of gut microbiota in metabolic disease [43]. Understanding these mechanisms has the potential to uncover new therapeutic targets for metabolic disorders.

Despite the insights provided, this study has several limitations. First, while the association between *Asic1a* deletion and altered gut microbiota composition is compelling, additional experiments and post‐fecal transplant sequencing are needed. Another limitation is the relatively short duration of the fecal microbiota transplantation study, which makes it difficult to determine whether the gut microbiota has long‐term effects on body weight gain. Due to the limited number of replicates and low statistical power, only descriptive results are reported. Second, the mechanistic pathways linking ASIC1a activity in the nervous system to peripheral metabolic regulation remain incompletely defined, especially in the gut. Third, the study was conducted in a rat model. While informative, it does not represent human physiology as there are no known disorders that directly result from ASIC mutation. While SNPs and regulatory variants in ASIC1a have yet to be linked to obesity and food intake, they are well known to affect neuropsychiatric conditions in population‐based studies [44, 45].

## 5 Conclusion

A key role of ASIC1a in regulating obesity and insulin resistance was uncovered, with these effects being further amplified with HFD administration. *Asic1a* deletion resulted in marked alterations to the gut microbiota, in a manner that likely contributes to insulin resistance and obesity progression. Overall, ASIC1a serves as a critical regulator of metabolic homeostasis, warranting further investigation. These results position ASIC1a as a molecular interface between the nervous system and peripheral metabolic regulation, with implications for the pathogenesis of obesity and related metabolic disorders.

## Author Contributions

C.M. and J.S. conceived the experiments. C.M. carried out the experiments, performed data analysis, and wrote the first draft of the manuscript. C.M. and O.E.‐U. carried out animal breeding. M.H.S. and A.C. helped develop the *Asic1a* knockout rats. J.A.T. provides resources for body composition measurements. K.D.M. and C.O. provided facility and resources for germ‐free studies. M.H.S. and J.S. secured the funding and had ultimate responsibility for the work. All authors reviewed the paper and approved of the submitted and published versions.

## Conflicts of Interest

The authors declare no conflicts of interest.

## Supporting information


**Figure S1:** Western blot confirmation of *Asic1a* knockout using brain cortex tissue.
**Figure S2:** Total AUC and incremental AUC of OGTT.
**Figure S3:** Food intake in male (A) and female (B) animals.
**Figure S4:** Effects of *Asic1a* deletion on blood biochemistry readouts and gut behavior. (A) Blood ketone at day 77. (B) Blood lactate at day 77. (C) Number of fecal pellets per rat at day 37. (D) Excreted fecal mass per rat at day 37. Two‐way ANOVA was applied to compare the effects of time and genotype. *n* = 2–7 per group.
**Figure S5:** Firmicutes to Bacteroidota ratio in feces. Statistical difference was analyzed by two‐way ANOVA.
**Figure S6:** Discriminant microbial features in males (A) and females (B). Statistical difference was analyzed with the linear discriminant analysis (LDA) effect size (LEfSe). LDA > 2 indicates significant features. Asic1a KO, acid‐sensing ion channels 1a knockout; CD, chow diet; WT, wild type.

## Data Availability

The data that support the findings of this study are available from the corresponding author upon reasonable request within 1 year of publication.
