# Supplementary material for: Role of Acid‐Sensing Ion Channels 1a in the Regulation of Obesity and the Gut Microbiota
Source: Obesity (Silver Spring). 2025 Oct 26;34(2):372–81. doi: 10.1002/oby.70059 (PMC12850573; doi:10.1002/oby.70059)
Supplement: Supplementary file 1 — Figure S1: Western blot confirmation of Asic1a knockout using brain cortex tissue. Figure S2: Total AUC and incremental AUC of OGTT. Figure S3: Food intake in male (A) and female (B) animals. Figure S4: Effects of Asic1a deletion on blood biochemistry readouts and gut behavior. (A) Blood ketone at day 77. (B) Blood lactate at day 77. (C) Number of fecal pellets per rat at day 37. (D) Excreted fecal mass per rat at day 37. Two‐way ANOVA was applied to compare the effects of time and genotype. n = 2–7 per group. Figure S5: Firmicutes to Bacteroidota ratio in feces. Statistical difference was analyzed by two‐way ANOVA. Figure S6: Discriminant microbial features in males (A) and females (B). Statistical difference was analyzed with the linear discriminant analysis (LDA) effect size (LEfSe). LDA > 2 indicates significant features. Asic1a KO, acid‐sensing ion channels 1a knockout; CD, chow diet; WT, wild type. [file OBY-34-372-s001.pdf]

Supplemental Materials

Role of acid-sensing ion channels 1a in the regulation of obesity and the gut microbiota

Jane Shearer[a,b,c], Morris H. Scantlebury[b,d,f,g], Oghenefejiro Erome-Utunedi[b,d], Anamika Choudhary[b,d,f], Jennifer A. Thompson[a], Christina Ohland[e], Kathy D. McCoy[e], Chunlong Mu[a,b,h]*

[a] *Department of Biochemistry and Molecular Biology, Cumming School of Medicine, University of Calgary, Calgary, AB T2N 1N4, Canada*

[b] *Alberta Children's Hospital Research Institute, University of Calgary, Calgary, AB T2N 1N4, Canada*

[c] *Faculty of Kinesiology, University of Calgary, Calgary, AB T2N 1N4, Canada*

[d] *Department of Pediatrics, University of Calgary, Calgary, AB T2N 1N4, Canada*

[e] *Department of Physiology and Pharmacology, Snyder Institute, Cumming School of Medicine,*

[f] *Hotchkiss Brain Institute, Cumming School of Medicine, University of Calgary, Calgary, AB T2N 1N4,*

[g] *Department of Clinical Neuroscience, Cumming School of Medicine, University of Calgary, Calgary, AB T2N 1N4*

[h] *Department of Microbiology, Immunology, and Infectious Diseases, Cumming School of Medicine, University of Calgary, Calgary, AB T2N 1N4, Canada*

Running Head: ASIC1a in Obesity

Keywords: Obesity, Insulin resistance, Acid Sensing Ion Channel, Gut microbiota

*For correspondence:

Dr. Chunlong Mu

Cumming School of Medicine

University of Calgary

3330 Hospital Drive NW

Calgary, Alberta T2N 4N1 Canada

Email: chunlong.mu1@ucalgary.ca

Conflicts of Interest: All authors have declared no conflict of interest exists.

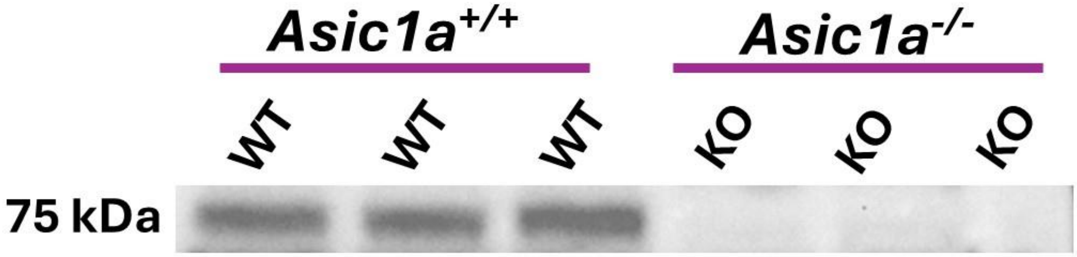

SUPPLEMENTAL FIGURE S1. Western blot confirmation of Asic1a knock out using brain cortex tissue.

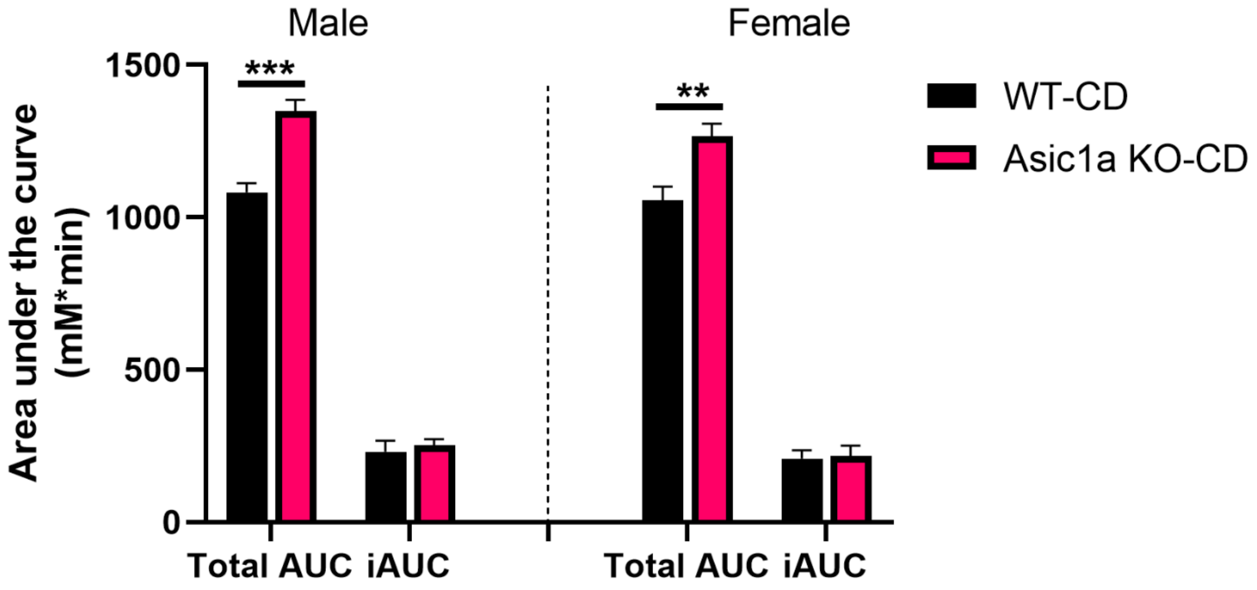

SUPPLEMENTAL FIGURE S2. Total AUC and incremental AUC of OGTT test.

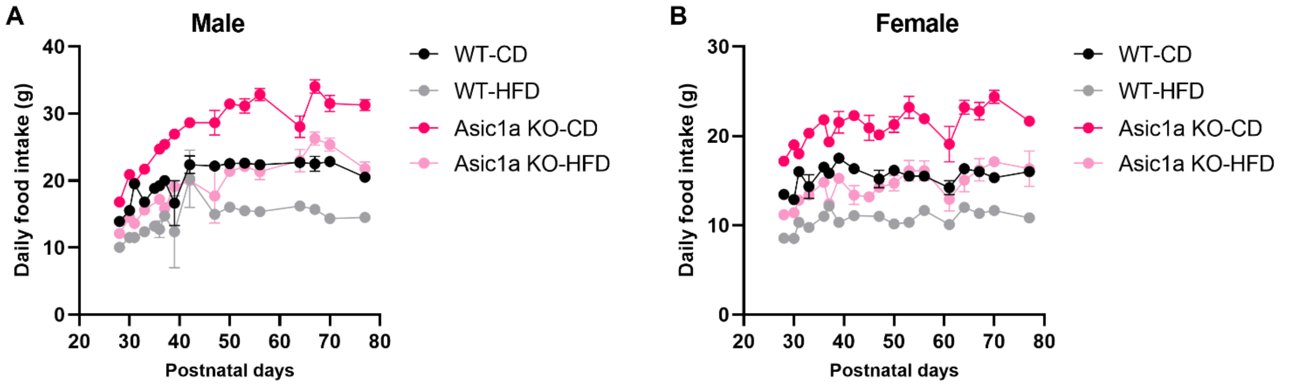

SUPPLEMENTAL FIGURE S3. Food intake in male (A) and female (B) animals.

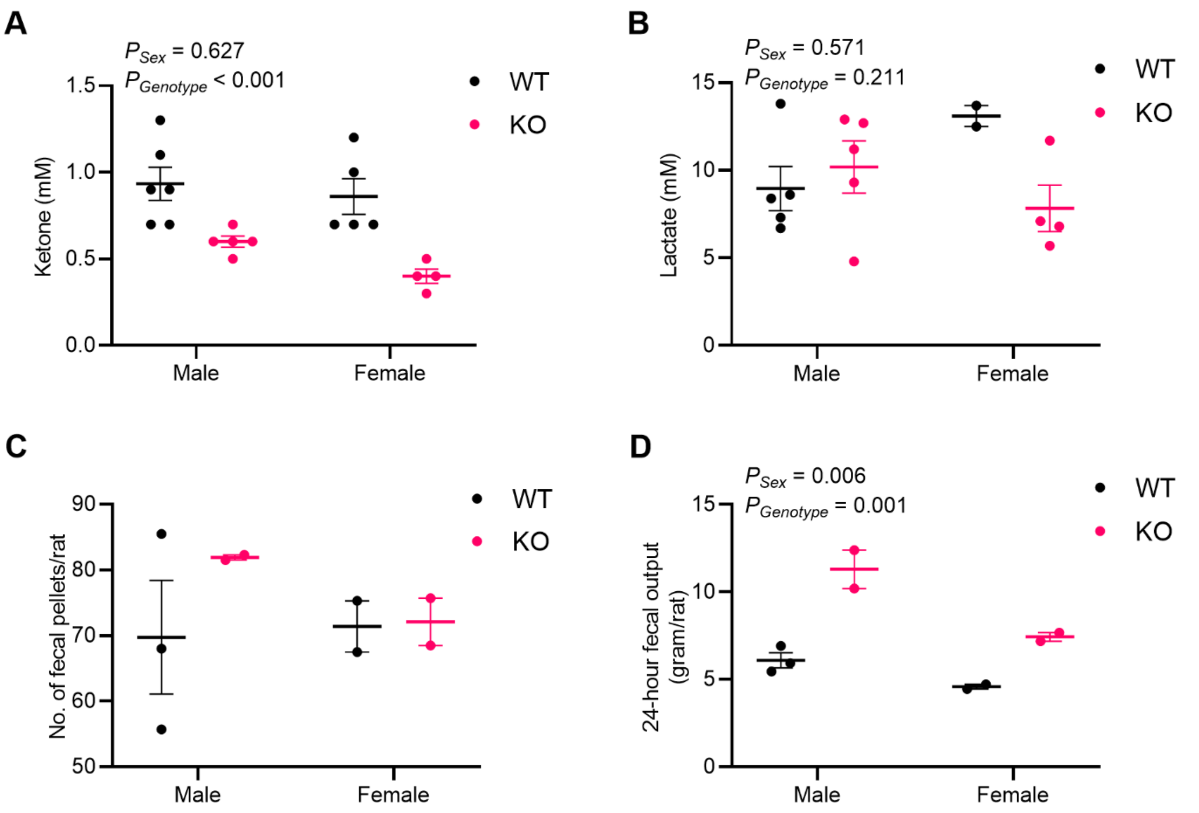

SUPPLEMENTAL FIGURE S4. Effects of Asic1a deletion on blood biochemistry readouts and gut behavior. (A) Blood ketone at day 77. (B) Blood lactate at day 77. (C) Number of fecal pellets per rat at day 37. (D) Excreted fecal mass per rat at day 37. Two-way ANOVA was applied to compare the effects of time and genotype. *n* = 2-7 per group.

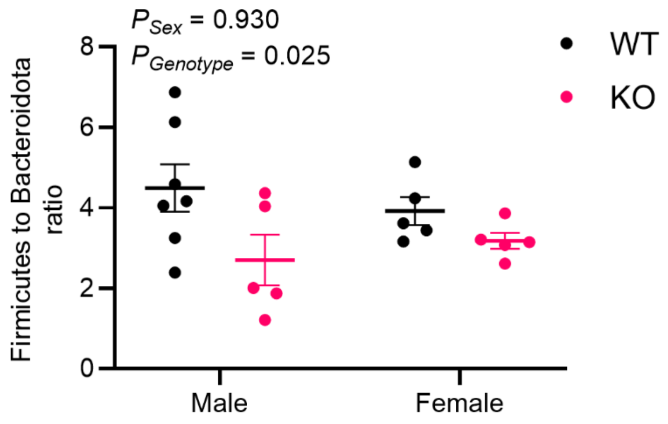

SUPPLEMENTAL FIGURE S5. Firmicutes to Bacteroidota ratio in feces. Statistical differences were analyzed by two-way ANOVA.

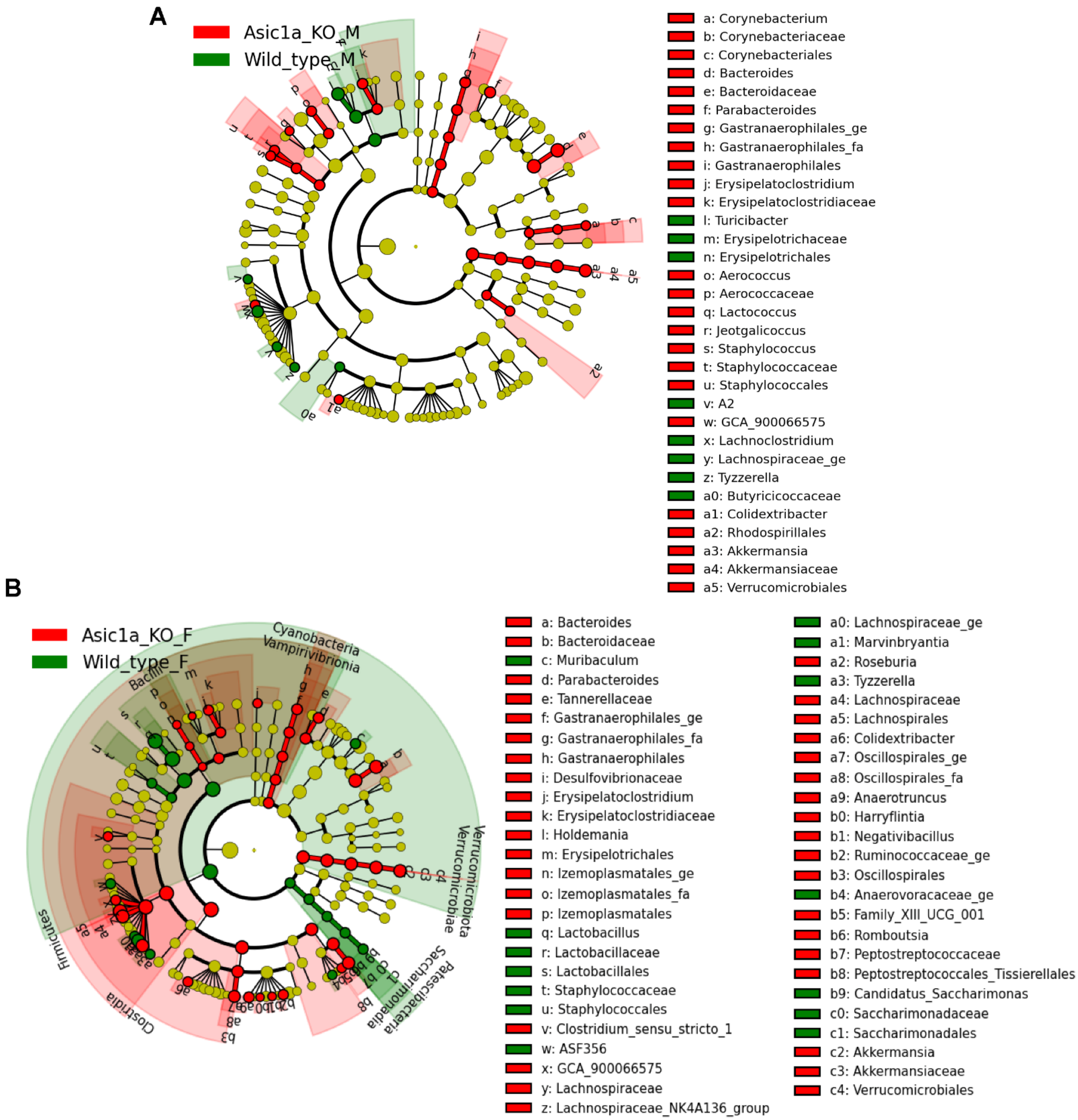

SUPPLEMENTAL FIGURE S6. Discriminant microbial features in males (A) and females (B). Statistical difference was analyzed with the linear discriminant analysis (LDA) effect size (LEfSe). LDA > 2 indicates significant features. Asic1a KO, Acid-sensing ion channels 1a knockout; CD, chow diet; WT, wild-type.
